# Natural Killer Group 2, Member D/NKG2D Ligands in Hematopoietic Cell Transplantation

**DOI:** 10.3389/fimmu.2017.00368

**Published:** 2017-03-27

**Authors:** Raphael Carapito, Ismail Aouadi, Wassila Ilias, Seiamak Bahram

**Affiliations:** ^1^ImmunoRhumatologie Moléculaire, INSERM UMR_S1109, LabEx TRANSPLANTEX, Centre de Recherche d’Immunologie et d’Hématologie, Faculté de Médecine, Fédération de Médecine Translationnelle de Strasbourg (FMTS), Université de Strasbourg, Strasbourg, France; ^2^Laboratoire International Associé (LIA) INSERM, Strasbourg (France) – Nagano (Japan), Strasbourg, France; ^3^Fédération Hospitalo-Universitaire (FHU) OMICARE, Strasbourg, France; ^4^Laboratoire Central d’Immunologie, Pôle de Biologie, Nouvel Hôpital Civil, Hôpitaux Universitaires de Strasbourg, Strasbourg, France

**Keywords:** natural killer group 2, member D, NKG2D ligands, hematopoietic cell transplantation, graft-vs.-host disease, MHC class I chain-related gene, MICA

## Abstract

Natural killer group 2, member D (NKG2D) is an invariant activatory receptor present on subsets of natural killer and T lymphocytes. It stimulates the cytolytic effector response upon engagement of its various stress-induced ligands NKG2D ligands (NKG2DL). Malignant transformation and conditioning treatment prior to hematopoietic cell transplantation (HCT) are stress factors leading to the activation of the NKG2D/NKG2DL signaling in clinical settings. In the context of HCT, NKG2D-bearing cells can kill both tumor and healthy cells expressing NKG2DL. The NKG2D/NKG2DL engagement has therefore a key role in the regulation of one of the most salient issues in allogeneic HCT, i.e., maintaining a balance between graft-vs.-leukemia effect and graft-vs.-host disease. The present review summarizes the current state of our knowledge pertaining to the role of the NKG2D and NKG2DL in HCT.

## Introduction

Hematopoietic cell transplantation (HCT) is a widely used curative treatment for a variety of both malignant and non-malignant hematological diseases (1). HCT involves the intravenous infusion of stem cells collected from bone marrow, peripheral blood, or umbilical cord blood, in order to reestablish hematopoietic function in patients whose bone marrow or immune system is damaged, defective or therapeutically ablated. Post-HCT reconstitution of the immune system is a crucial step for remission and involves, among other immunological cells, cytotoxic lymphocytes. Because of their ability to selectively kill malignant or infected cells, these lymphocytes are key players in immune surveillance. They include natural killer (NK) and T cells, which share the expression of the activatory *natural killer group 2, member D* (NKG2D) receptor. While important for killing malignant cells [graft-vs.-leukemia (GVL) effect] and protecting the immunosuppressed patient undergoing HCT against opportunistic infections, these cells can also provoke adverse outcomes where graft-vs.-host disease (GVHD) is the most dramatic.

Natural killer group 2, member D is a C-type lectin-like type II transmembrane protein encoded by the “*killer cell lectin-like receptor K1*” (*KLRK1*) gene, embedded within the NK-gene complex on human chromosome 12. It functionally assembles into a hexameric structure where each NKG2D monomer is associated with a DNAX-activating protein 10 (DAP10) dimer in man, an adapter protein bearing a YXXM motif that recruits phosphoinositide 3-kinase. The NKG2D receptor is present on NK cells, CD8^+^ αβ, subsets of CD4^+^ αβ, as well as γδ T lymphocytes as well as NKT cells (2, 3). It is a genetically invariant (very few alleles have been reported) receptor that stimulates effector responses upon engagement of various stress-inducible ligands; hereafter called NKG2D ligands (NKG2DL). The function of NKG2D is therefore directly linked to the biology of its ligands, which expression is absent or low in most “normal” cells, but induced by cell stress, whether caused by infection, malignant transformation, or conditioning treatments prior to HCT (3–5).

In human, there are two families of NKG2DL: the “*MHC class I chain-related genes*” (*MIC*) and “*UL16-binding proteins*” (*ULBPs*) genes; the latter also known as retinoic acid early transcripts 1 (*RAET1*). The first family includes the two non-conventional MHC-I loci *MICA* and *MICB* encoded within the MHC; at the centromeric extremity of the MHC class I region (6). These genes encode highly glycosylated MHC class I-like proteins, which are expressed at the cell surface independently of β_2_-microglobulin and cytosolic peptides. The genomic and protein structure of *MICA* and *MICB* is similar to those of conventional MHC class I genes and molecules. The proteins are composed of three extracellular domains (α_1_, α_2_, and α_3_), a transmembrane region, and a cytoplasmic tail. Unlike other non-conventional MHC class I genes, a high degree of diversity has been documented for *MICA* and *MICB* genes: 106 alleles for *MICA* and 42 for *MICB* thus far (http://hla.alleles.org/data/index.html). *MICA* polymorphism has been associated with a number of diseases such as autoimmune disorders (7, 8) and cancer (9, 10), but also with allograft rejection and GVHD (11–16). The second NKG2DL family encompasses six functional genes: *RAET1I/ULBP1, RAET1H/ULBP2, RAET1N/ULBP3, RAET1E/ULBP4, RAET1G/ULBP5*, and *RAET1L/ULBP6*. These genes are also localized on chromosome 6, but on its opposite arm with respect to the MHC, between cytogenetics bands 6q24.2 and 25.3. In contrast to MIC proteins, all ULBP/RAET1s lack an α_3_ domain, and *RAET1I/ULBP1, RAET1H/ULBP2, RAET1N/ULBP3*, and *RAET1L/ULBP6* are attached to the membrane *via* a glycosylphosphatidylinositol anchor. *ULBP/RAET1* genes appear to be less polymorphic as *MICA* and *MICB*. This may, however, not reflect the reality as only about 300 individuals have been sequenced thus far. In mice, there are three families of NKG2DL: *Rae-1/RAET1* (five known proteins), *H60* (three known proteins), and *MULT-1* (one known protein) but as evidenced early-on no orthologous *MIC* genes (17).

Several converging lines of evidences indicate that the NKG2D–NKG2DL interaction is a key event in the regulation of the immune response following HCT, especially with respect to GVHD and GVL effect. First, NKG2DL proteins are mainly expressed in cells of fibroblastic and epithelial origin (18), which is in accordance with the localized tissues expression of GVHD (skin, liver, and gut). Second, in line with a possible involvement in the GVL effect, NKG2DL are known to be upregulated in tumor cells (19–21). Third, the systemic inflammatory response to conditioning regimens can serve as a danger/stress signal, which is needed to induce NKG2DL expression (22). Fourth, the DNA damage pathway that is activated in response to ionizing radiation and chemotherapy is central in the upregulation of NKG2DL (23). Fifth, neutralization of the NKG2D receptors by antibodies prevents graft rejection in mice (24, 25). Finally, blockade of the NKG2D/NKG2DL interaction by antibodies directed against NKG2D, attenuate GVHD while allowing CD8^+^ T cells to regain their GVL activity (26).

Here, we review the current knowledge of the role of both NKG2D and NKG2DL in HCT at the levels of genetic polymorphism, protein expression regulation, and antibody production.

## Genetic Polymorphism of NKG2D and NKG2DL

Both human NKG2DL gene families have been studied with respect to genotype–phenotype relationship in the context of HCT. While several recent publications have analyzed the influence of *MIC* genes in HCT, a single study focused on the role of the *ULBP/RAET1* gene family. In this study, Antoun and coworkers used a cohort of 371 patient/donor pairs of HLA-matched related allografts to analyze a total of 18 single nucleotide polymorphisms (SNPs) in the four most polymorphic members of this gene family, i.e., *RAET1N/ULBP3, RAET1E/ULBP4, RAET1G/ULBP5*, and *RAET1L/ULBP6* (27). The only gene that could be related to clinical outcomes was *RAET1L/ULBP6*. Thanks to SNP haplotype structure analysis of this gene, the authors could associate the presence of the RAET1L*02 allele in patients with an improved overall survival (55% in patients with RAET1L*02 vs. 39% in patients without RAET1L*02, P = 0.003) and relapse-free survival (44% in patients with RAET1L*02 vs. 25% in patients without RAET1L*02, P < 0.001). In addition, this allele was found to be associated with a reduced risk of relapse, with 11.5% of risk for RAET1L*02 homozygotes compared to 29.1% for RAET1L*01 heterozygotes and 38.2% for the RAET1L*02 negative patients (*P* = 0.001 between the homozygous groups). Of note, neither the identity, nor the matching of the donors’ alleles had an impact on clinical outcomes.

With regards to *MIC* genes, in the last decade several genetic studies have collectively concluded to a potential role of MICA as a novel transplantation antigen. The very first hint toward a significant role of *MICA/B* matching in HCT came from an MHC “beta block” matching study (28). In this work, a small cohort of 44 donor/recipient pairs of unrelated allogeneic HCT was used to determine the correlation of the so-called “beta block”—*HLA-B, -C, MICA*, and *MICB*—matching on patient survival. The “beta block” spans 300 kb, harbors the immunology-related genes *HLA-B, HLA-C, MICA*, and *MICB* genes, and represents one of the four “MHC blocks” including also the alpha (cluster around *HLA-A*), gamma (cluster around *Bf* and *C4*), and delta (cluster around *HLA-DR* and *-DQ*) blocks (29). Patients who were *HLA-B* and *-C* matched showed an increased survival when they were additionally matched for the MHC beta block (59 vs. 16%, *P* = 0.04) or *MICA* and *MICB* (66 vs. 25%, *P* = 0.05). In addition, as patients who were beta block matched were matched at both *HLA-B* and *MICB* but not *HLA-C* and *MICA*, the authors could show that in this subset of patients, *MICA*- and *HLA-C*-matched patients had a significantly improved survival of 69% (*P* = 0.05).

Other studies have specifically analyzed the influence of the *MICA* gene by three different approaches: (i) focused analysis of the genotype of a single polymorphism at amino acid position 129 in patients (12, 15), (ii) matching of donors and patients genotypes at the same position; 129 (14), and (iii) matching of donor and patients at the *MICA* allele level (13, 16, 30, 31).

The polymorphism at amino acid position 129 involving a substitution of a methionine for a valine is known to lower the binding affinity of MICA for NKG2D, probably through a conformational change since this position is not directly involved in NKG2D binding (32–34). Patients’ genotype at position 129 was firstly analyzed in a cohort of 211 matched related allogeneic HCT patients, and it was shown that those bearing the MICA-129 Val/Val genotype—i.e., the lower binding affinity variant—were at a higher risk of developing chronic GVHD (63 vs. 45% at 3 years; *P* = 0.03) (12). Moreover, the relapse incidence in patients with the MICA-129 Met/Met genotype was higher than in those with other genotypes (51 vs. 19%, *P* = 0.007). A second group analyzed 452 patients that underwent matched unrelated (67.9% of patients) and related (31.6% of patients) allogeneic HCT and evidenced that the patients with the MICA-129 Met genotype had an increased probability of overall survival (HR = 0.77, *P* = 0.0445) and that the mortality specifically due to acute GVHD was reduced (HR = 0.57, *P* = 0.0400) (15). The improved survival of MICA-129 Met carriers was also observed in the subset of patients that received an MICA-129-matched transplant (*n* = 404, HR = 0.73, *P* = 0.0226). However, this study unexpectedly also reported an increased risk of acute GVHD (HR = 1.92, *P* = 0.0371) in MICA-129 Met/Met homozygote carriers, i.e., the higher binding affinity variant. As this was particularly the case for anti-thymocyte globulin (ATG) treated patients who have a lowered CD8^+^ T repertoire, the authors argued that the residual T CD8^+^ cells may be activated faster in the presence of two MICA-129 Met variants. In the case of heterozygote carriers, the beneficial effect was indeed attributed to the subgroup of patients not treated with ATG, indicating that such a faster activation may not matter if a full CD8^+^ T cell repertoire is present. Finally, a third publication by Askar et al. reported that neither the patient’s nor the donor’s genotype at position 129 had an impact on survival or GVHD (30). The authors could only detect an association between donor MICA-129 non-Val/Val genotypes and slower platelet engraftment (HR = 1.4; 95% CI: 1.109–1.985; *P* = 0.02).

More recently, a group analyzed not only the MICA-129 genotype of the patient but also the one of the donor and by this mean the role of the matching of donor and recipients at this position with respect to various clinical outcomes after unrelated HCT (14). They analyzed 2,172 patients that underwent unrelated HCT with donors HLA-matched at various grades (10/10, 9/10, and 8/10). They showed that matching of MICA-129 genotypes between donors and patients was important to increase overall survival and disease free survival and to lower the risk for acute GVHD. In the 10/10 matched group, multivariate analysis revealed indeed a clear association of MICA-129 mismatches with overall survival (HR = 1.77, 95% CI: 1.22–2.57, *P* = 0.003), disease free survival (HR = 1.77, 95% CI: 1.26–2.50, *P* = 0.001), and acute grade III–IV aGvHD (prevalence of 16.3 vs. 11.0%).

Finally, the third type of analysis was to consider, as it is done for classical HLA genes, the matching of donors and recipients at the MICA allele level. By analyzing a cohort of 172 matched pairs of unrelated HCT, Parmar et al. showed a higher rate of grade II–IV acute GVHD in *MICA*-mismatched vs. -matched patients, taking only account of *MICA* mismatches in the GVHD direction (75 vs. 39%, *P* = 0.02) (16). Askar and coworkers also studied an equally small cohort of 177 10/10 matched donor–recipient pairs (31). They did find a link between *MICA* mismatches and grade II–IV acute GVHD but in univariate analysis only; this link was reenforced in patients with mismatches at both *MICA* and *HLA-DPB1* who had a significantly greater risk to develop grade II to IV acute GVHD (HR = 2.51; 95% CI: 1.30–4.87; *P* < 0.01). More recently, we were able to definitely establish the role of *MICA* mismatches in HCT based on the analysis of a multicenter cohort of 922 10/10 HLA-matched HCT. *MICA* mismatches were significantly associated with an increased incidence of grade III–IV acute GVHD (HR = 1.83; 95% CI: 1.50–2.23; *P* < 0.001), chronic GVHD (HR = 1.50; 95% CI: 1.45–1.55; *P* < 0.001), and non-relapse mortality (HR = 1.35; 95% CI: 1.24–1.46; *P* < 0.001). The increased risk of GVHD was mirrored by a lower risk of relapse (HR = 0.50; 95% CI: 0.43–0.59; *P* < 0.001), indicating a possible GVL effect. A few weeks later in 2016, Askar and coworkers, however, published contradictory results using a cohort of 713 patients and their unrelated HLA-matched 10/10 (*n* = 552) or 9/10 (*n* = 161) donors (30). They showed an absence of MICA mismatch effect on all major clinical outcomes apart an unexpected significantly higher incidence of relapse in *MICA*-mismatched vs. -matched patients (HR = 1.7; 95% CI: 1.2–2.4; *P* = 0.003). In their data, there was, however, a clear trend for an association between *MICA* mismatches and a higher risk of acute GVHD grades II–IV (HR = 1.4; 95% CI: 1.1–1.9; *P* = 0.013). The various genetic studies on *NKG2D/NKG2DL* polymorphisms or donor/recipient matching in HCT and their main findings are summarized in Table 1.

**Table 1:** **Summary of genetic studies of natural killer group 2, member D (NKG2D)/NKG2D ligands polymorphisms or donor/recipient matching in hematopoietic cell transplantation (HCT)**.

Effect	Gene	Alleles/genotypes	Number and type of transplantation^a^	Reference
Risk of aGVHD	*MICA*	*MICA*-129 Met/Met in recipients	452 UD	(15)
	*MICA*	*MICA* donor–recipient mismatches^b^	236 UD	(16)
	*MICA*	*MICA*-129 mismatches	2,172 MUD	(14)
	*MICA*	*MICA* donor–recipient mismatches	922 MUD	(13)
	*MICA*	*MICA* donor–recipient mismatches^c^	713 MUD	(30)
	*MICA* and *HLA-DPB1*	*MICA* and HLA-DPB1 donor–recipient mismatches	227 UD	(31)
Risk of cGvHD	*MICA*	*MICA*-129 Val/Val in recipients	211 MRD	(12)
	*MICA*	*MICA* donor–recipient mismatches	922 MUD	(13)
Improved overall survival	*MICA*	*MICA* donor–recipient matches^b^	922 MUD	(13)
	*MICA* and *MICB*	*MICA* and *MICB* donor–recipient matches	44 UD	(28)
	*NKG2D*	rs1049174—HNK1 haplotype positive in donors	145 MUD	(43)
	*MICA*	*MICA*-129 matches	2,172 MUD	(14)
	*MICA*	*MICA*-129 Met/Val or Met/Met in recipients	452 UD	(15)
	*RAET1L/ULBP6*	RAET1L*02 in patients	371 MRD	(27)
Decreased non-relapse mortality	*MICA*	*MICA* donor–recipient matches	922 MUD	(13)
	*NKG2D*	rs1049174—HNK1 haplotype positive in donors	145 MUD	(43)
Improved disease free survival	*MICA*	*MICA*-129 matches	2,172 MUD	(14)
	*RAET1L/ULBP6*	RAET1L*02 in patients	371 MRD	(27)
Reduced risk of relapse	*MICA*	*MICA*-129 Met/Val or Val/Val in recipients	211 MRD	(12)
	*MICA*	*MICA* donor–recipient matches	713 MUD	(30)
	*MICA*	*MICA* donor–recipient mismatches	922 MUD	(13)
	*RAET1L/ULBP6*	RAET1L*02 homozygous in patients	371 MRD	(27)

*^a^Types of transplantations are unrelated donor (UD), matched unrelated donor (MUD), and matched-related donor (MRD) HCT*.

*^b^Only mismatches in the GVH direction were considered*.

*^c^Trend for association (*P* = 0.013)*.

**Figure 1:**
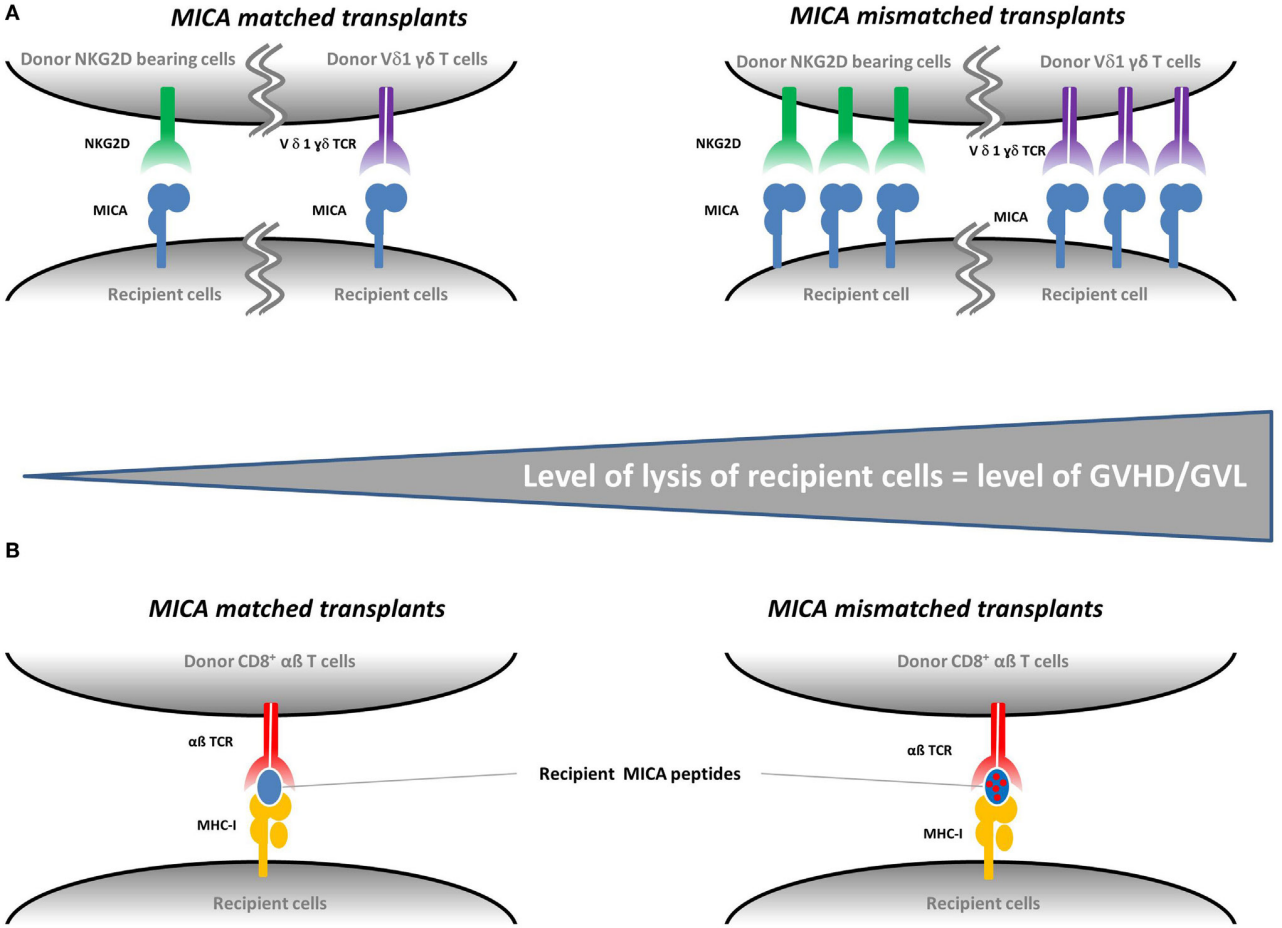
**Proposed mechanisms for the role of *MICA* mismatches in graft-vs.-host disease (GVHD)**. There are two non-exclusive possible molecular mechanisms involved in the development of GVHD in the context of *MICA*-mismatched transplants. Panel **(A)** illustrates how an increased MICA expression—possibly induced by *MICA* mismatches—could lead to an increased cytolysis of recipient cells mediated by the natural killer group 2, member D (NKG2D) on donor natural killer cells and/or the TCR of donor Vδ1-bearing γδ T cells and/or donor T CD8^+^ cells as a co-stimulatory function. GVHD and graft-vs.-leukemia (GVL) can be triggered if the target cells of the recipients are healthy or malignant, respectively. Panel **(B)** illustrates how *MICA* could act as a minor histocompatibility antigen. If the donor is matched for *MICA*, the recipient MICA-derived peptides are recognized as «self» by donor cells. If the donor is mismatched for *MICA*, the presented recipient MICA-derived peptides are recognized as «non-self» and recipient cells are lysed by donor CD8^+^ T cells.

Globally, these findings clearly support a role of MICA as a transplantation antigen in HCT. Concerning the pathophysiological explanation of the observed associations, there are two non-exclusive possible molecular mechanisms that could be involved. Integrating the fact that MICA expression is upregulated in intestinal tissues of GVHD patients and that NKG2D is induced on CD8^+^ and CD56^+^ cells after HCT (see next section); it is likely that a mismatch between donor/recipient MICA molecules directly affects the strength of recognition/signaling and hence cytotoxicity by NKG2D bearing T/NK cells and/or the intestine-enriched Vδ1-bearing γδ T cells (both receptors able to recognize MICA directly). While the direct link between *MICA* mismatches and increased expression needs to be experimentally demonstrated, two groups reported variable NKG2D-mediated NK-cell activation and T CD8^+^ co-stimulation depending on specific *MICA* alleles. Tonnerre et al. showed a higher NK-cell activation by allogeneic endothelial cells expressing the *MICA A5.1* alleles (35), and Isernhagen et al. demonstrated an increased CD8^+^ T cell co-activation in response to the MICA-129 Met variant (15). Alternatively, MICA could act as a minor histocompatibility antigen, i.e., a source for polymorphic peptides presented by cognate and/or donor MHC class I molecules and hence participate in the GVHD pathophysiology through its contribution to alloreactivity. These two possibilities are schematized in Figure 1. Of note, NKG2D engagement by MICA could also play an indirect role in the enhancement of CD8^+^ T cytolysis, which is known to elicit GVHD (1). Although it has been shown that NKG2D may trigger cytotoxicity alone in expanded CD8^+^ T cells, cultured in the presence of IL-2 (36), several studies in human and in mouse reported that NKG2D rather acts as a co-stimulatory molecule in CD8^+^ T cells (26, 37–40). In T cells, NKG2D indeed associates with an adaptor protein, DAP10, where it provides a co-stimulatory signal by activating intracellular signaling pathways (37, 40, 41). By providing T cell co-stimulation, NKG2D ligand engagement may therefore enhance CD8^+^ T cell-mediated cytotoxicity of recipient cells, thereby triggering GVHD (42).

To conclude this section, a single study on NKG2D receptor polymorphism has to be mentioned. As mentioned above and in contrast to its ligands, NKG2D is not polymorphic. Only two SNPs with a minor allele frequency >0.01 have been reported in the coding sequence of the gene: the synonymous rs1049172 and the non-synonymous rs2255336. But the most studied SNP is the rs1049174 in the 3′-untranslated region of the gene, which enables to distinguish two haplotypes: HNK1 (high NK cytotoxicity) and LNK1 (low NK cytotoxicity). The HNK1 haplotype has been associated with improved overall survival (HR = 0.44; 95% CI: 0.23–0.85; *P* = 0.01) as well as transplant related mortality (HR = 0.42; 95% CI: 0.21–0.86; *P* = 0.02) after unrelated HLA-matched unrelated HCT (43).

## Upregulation of NKG2D and NKG2DLs

Besides the role of donor/recipient compatibility detailed in the previous chapter, the stress-induced upregulation of NKG2DLs in patient’s tissues may *per se* contribute to the development of GVHD. In contrast to solid organ transplantation, where it is established that MIC antigens are expressed in transplanted organs and may cause early graft rejection (44, 45), only few studies analyzed NKG2DL protein expression in the context of HCT. By immunochemical staining, Dulphy et al. showed a strong expression of MICA on epithelial cells of intestinal biopsies from acute GVHD patients (46). Gannagé et al. confirmed this observation and also demonstrated that during GVHD, MICA/B expression was induced in skin and liver biopsies as well (5). In addition, they showed that *in vitro*, in immortalized human keratinocytes and colorectal carcinoma fibroblasts both MICA/B and ULBP1–3 were upregulated in response to TNF-α or γ-irradiation. Total body irradiation that is regularly used in HCT could therefore be a trigger for NKG2DL expression. But other types of pretransplant treatments such as chemotherapies could also play this role, e.g., the DNA-hypomethylating drug 2-deoxy-5-azacytidine (decitabine), which has been shown to upregulate MICB in cell lines (22). Finally, cancer cells by themselves have a propensity for NKG2DL upregulation. This was demonstrated by Sconocchia and coworkers who found an MICA/B overexpression on chronic myelogenous leukemia CD34 cells but not on normal CD34 cells and showed that this upregulation correlated with the ability of soluble MICA (sMICA)/B to bind to the NKG2D receptor (47).

The latter mentioned soluble form of NKG2DLs corresponds to a well-known immune escape mechanism in cancer cells. NKG2DLs and especially MICA have indeed the ability to be released from the cell surface through various mechanisms including alternative splicing, phosphatidylinositol-specific phospholipase C-mediated cleavage, proteolytic shedding, or exosome secretion [reviewed in Ref. (17)]. The soluble form of NKG2DL downregulates NKG2D and thereby impairs the cytolysis activity of NK and T effector cells (48). By doing so, tumor cells evade immune surveillance (48–50). Soluble MIC and ULBP proteins have been identified in the sera of patients with various tumor types including breast, lung, colon, and ovarian carcinoma, glioma, neuroblastoma, leukemia, and melanoma (48, 50–55). Following HCT, the serum levels of sMICA have been shown to increase and the presence of these molecules to confer susceptibility to chronic GVHD (sMICA > 80 pg/mL associated with chronic GVHD incidences of 82 vs. 46%, at 3 years; *P* = 0.001) (12, 56). Opposite to this increase of the GVH effect on recipient cells, high levels of sMICA were shown to decrease the GVL effect as shown by the higher rate of relapse (sMICA > 80 pg/mL associated with relapse rates of 37 vs. 17%, *P* = 0.05) (12). This trend toward a lowered cytolytic activity against cancer cells was confirmed in solid tumors (prostate cancer), where a higher level of sMICA has been associated with a decreased cytotoxicity of NK cells isolated from tumor biopsies (57).

The molecular mechanisms of expression regulation of NKG2DLs are still poorly understood. However, experimental data on two pathways are available: the DNA damage response and regulation by cellular and viral miRNA. Data from both mice and human indicate that NKG2DL are induced by DNA damaging agents such as 5-aza-2′-deoxycytidine (decitabine), a conditioning treatment used in myelodysplastic syndromes (22, 23, 58). The upregulation is thought to be due to promoter DNA demethylation in combination with the activation of the DNA damage pathway involving the protein kinases ataxia telangiectasia mutated (ATM) and ATM- and Rad3-related (23). For miRNA regulation, both viral (*Cytomegaloviru*s) and cellular miRNA have been shown to modify the expression of various NKG2DLs and thereby to influence NK-cell responses (59–62).

For the ligand expressing cells to be efficiently targeted and killed, their receptor on effector cells must be expressed and active as well. Although the role of NKG2D in tumor immunity is not to be proven anymore as shown by the multiple studies demonstrating its involvement in NK cell-mediated killing of various cancer cells (63–65), its specific action in the context of HCT has only been scarcely analyzed. Only a handful of studies have indeed reported active NKG2D upregulation during HCT. Lu et al. firstly observed an increase in CD16^+^CD56^−^ NK cells in peripheral blood of a cord blood transplanted patients (66). When cultured *in vitro* in presence of IL-2, these cells became CD16^+^CD56^+^NKG2D^+^ and were able to lyse the patient’s ULBP2-expressing leukemic cells. These results indicate that *in vivo*, after transplantation, mature NK cells derived from this NK-cell subset may contribute to the killing of leukemic cells expressing NKG2DL. Dulphy et al. also showed an expansion of CD56^bright^ NK cells shortly after HLA matched related and unrelated HCT (46). NKG2D was expressed on these cells, which were also shown to be functional as they produced high amounts of IFN-γ and were competent for degranulation. In line with these two reports, Picardi et al. showed an upregulation of NKG2D on CD8^+^ and/or CD56^+^ cells between 30 and 90 days posttransplant in HLA-matched allogeneic and autologous HCT, coinciding with the engraftment period (67). The authors suggest that this upregulation, together with a concomitant upregulation of inhibitory NKG2 receptors, is the sign of an immune “reeducation” and a general stress response to pretransplant chemotherapy and posttransplant repopulation. The regulation of NKG2D expression is thought to be modulated by cytokines such as IL-15, which ultimately control the levels of NK cytotoxicity (68). Of note, one study reports that NKG2D expression by NK cells was not affected after fully haplo-mismatched-related donor HCT, challenging the importance of the interaction between NKG2D and its ligands for the NK lysis of leukemic cells at least in this transplant setting (69).

Natural killer cells are the first lymphocyte population to be reconstituted following HCT and are important in mediating the GVL effect (70). NK cells could be subtyped into three categories based on the following surface makers: CD56^dim^/CD16^+^ (hereafter termed CD56^dim^), CD56^bright^/CD16^+/−^ (hereafter termed CD56^bright^) and CD56^−^/CD16^+^ (hereafter termed CD56^neg^) NK cells. The CD56^dim^ cells represent up to 90% of circulating NK cells and are considered as the most cytotoxic subset. The CD56^bright^ subtype comprises up to 10% of circulating NK cells but is the major NK subtype in tissues and secondary lymphoid organs. This subset is conventionally known as the cytokine-producing subset of NK cells but can also become cytotoxic upon appropriate activation. CD56^neg^ NK cells are found in small numbers in healthy individuals and at elevated levels in individuals chronically infected with HIV-1 and HCV. All three subsets of NK cells express NKG2D (71, 72). After allogeneic HCT, there is an early expansion of the cytokine-producing CD56^bright^ NK-cell subset, followed by an expansion of the dominant CD56^dim^ subset, characterized by its higher cytotoxic activity (46). In a study focusing on the immune-regulatory role of NK cells, it was shown that NKG2D plays a major role in NK cell-mediated lysis of activated CD4^+^ T cells. Using anti-NKG2D antibodies, the authors evidenced equivalent NKG2D-mediated degranulation by CD56^dim^ and CD56^bright^ NK cells (73). In ovarian cancer patients, it was shown that CD56^bright^ NK cells had increased expression of NKG2D (74). Finally, CD56^neg^ NK cells were shown to be increased in cord blood recipients, suggesting that this subset may contribute to the killing of NKG2D expressing leukemic cells (66).

**Figure 2:**
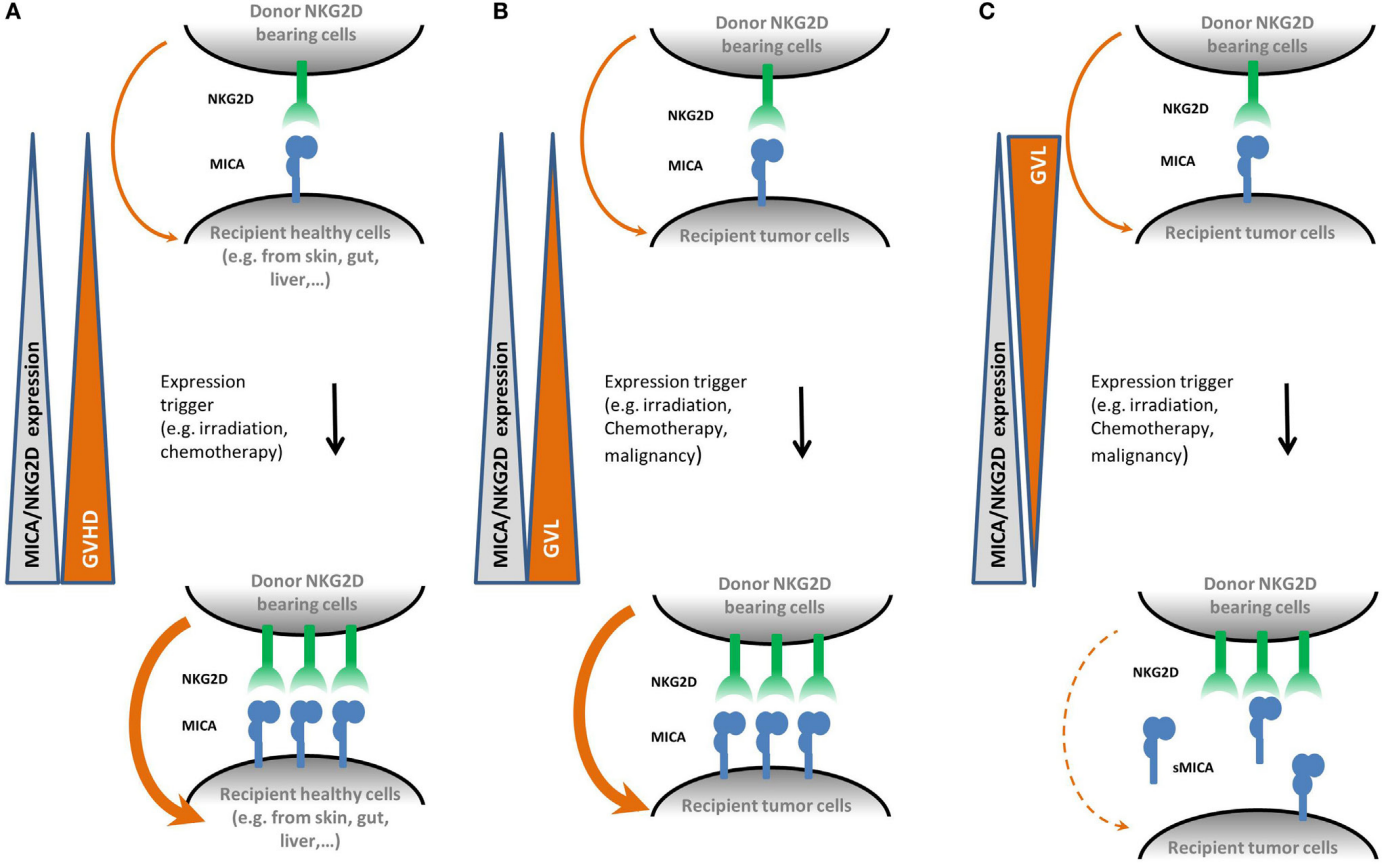
**Proposed mechanisms for the role of natural killer group 2, member D (NKG2D) and MICA upregulation in graft-vs.-host disease (GVHD) and graft-vs.-leukemia (GVL) effect mediated by natural killer (NK) cells**. Panel **(A)** shows that following hematopoietic cell transplantation (HCT) conditioning regimen, healthy recipient cells upregulate membrane-bound MICA expression. As NKG2D is known to be upregulated during HCT as well, it could therefore participate to an increased cytolysis activity alone or as a co-stimulatory signal on donor T CD8^+^ cells, thereby promoting the development of GVHD. Panel **(B)** shows the equivalent situation on recipient cancer cells. Here, upregulation of membrane bound MICA expression promotes the GVL effect. Panel **(C)** illustrates the effect of soluble MICA (sMICA) production by recipient cancer cells. sMICA leads to the downregulation of NKG2D and thereby to an impairment of the cytolysis activity of NK effector cells, i.e., lower the GVL effect.

The function of NK cells is modulated by the balance between a number of activating (including NKG2D) and inhibitory receptors. NKG2 and killer immunoglobulin-like receptors (KIRs) are the two main receptor families involved in this regulation and both encompass activatory and inhibitory receptors. KIR receptors interact with HLA class I molecules and, in contrast to NKG2D, are very diverse with regard to gene polymorphism and content, expression level, and expression pattern (75). In the context of HCT, KIR–ligand mismatches in the GVH direction were shown to trigger donor-vs.-recipient NK-cell alloreactivity (76). Overall, together with other receptors such as immunoglobulin-like receptors or leukocyte-associated immunoglobulin-like receptor, KIR and NKG2 receptors regulate the alloreactivity of NK cells during HCT (77). Altogether, these data strongly suggest that the induction of the NKG2D/NKG2DL axis might be involved in tissue damage during GVHD or GVL reactions thanks to NKG2DLs and NKG2D expression by host or cancer cells, respectively. A summary of the impacts of NKG2DL and NKG2D upregulation during HCT is presented in Figure 2.

## Anti-MICA Antibodies

In the field of transplantation, studies on antibodies against NKG2DL have so far exclusively focused on MICA. The presence of anti-MICA antibodies is a well-known risk factor for both acute and chronic rejection after transplantation of kidney (78, 79), pancreas (45), or heart (80). In HCT, three groups have analyzed the role of anti-MICA antibodies. Boukouaci et al. showed that the presence of anti-MICA antibodies with concomitant low serum levels of sMICA (<80 pg/mL) was associated with a lower incidence of chronic GVHD (35% when MICA antibodies positive and sMICA < 80pg/mL vs. 81% when MICA antibodies negative or borderline and sMICA > 80 pg/mL, *P* = 0.0004) (12). The authors hypothesized that these antibodies have a neutralizing activity on sMICA and supported this theory by the observed trend for a protective effect of anti-MICA antibodies against relapse (3.26% when MICA antibodies negative vs. 6% when MICA antibodies positive, *P* = 0.05). In a second study focusing on a cohort of 70 pediatric patients that received cord blood transplantation, Ansari et al. showed that the presence of anti-MICA antibodies was associated with a reduced platelet recovery after transplantation (26.5% when MICA antibodies negative vs. 80.5% when MICA antibodies positive, *P* = 0.04) (81). Finally, Flaxa et al. could observe immunization against MICA before and after transplantation in a small series of eight granulocyte transfused patients that underwent HCT, but those antibodies did not significantly affect overall survival or the incidence of GVHD (82).

In summary, in contrast to solid organ transplantation very limited MICA immunization data are available for HCT. Thus, at this stage, any definitive conclusion on the role of anti-MICA or more generally anti-NKG2DL antibodies in HCT cannot be drawn without performing further experiments. Several studies, however, confirm that such antibodies could be involved in GVL. Jinushi et al. demonstrated that MICA antibodies can efficiently opsonize and participate in the complement-mediated lysis of cancer cells (83). In a follow-up study, the same group showed that in monoclonal gammopathy of undetermined significance patients, high levels of anti-MICA antibodies antagonize sMICA and stimulate dendritic cell cross-presentation of tumor antigens (49). This observation had previously been made Groh et al. in an *in vitro* assay: by loading dendritic cells with anti-MICA opsonized breast, melanoma, or ovarian tumor lines, tumor antigen cross-presentation was promoted and thereby primed antitumor effector CD4 and CD8 T cell responses (84).

In the field of organ transplantation, the production of anti-MICA antibodies has been associated with donor/recipient *MICA* mismatching (85). Another study showed that the genetic variant *MICA A5.1* (a microsatellite marker in exon 5 encoding the transmembrane region of the protein) increased the surface expression of MICA and augmented the release of soluble and exosomal forms of MICA from endothelial cells (35). In addition, the authors demonstrate a significant association between donor/recipient *MICA A5.1* mismatches and anti-MICA allo-immunization, particularly when donors carry the MICA *A5.1* variant (*P* = 0.0104). In other fields of research such as cancer and infectious diseases, several *MICA* polymorphisms have been reported to affect MICA shedding including an SNP (rs2596542) in the promoter region (86–89), the microsatellite *MICA A5.1* (35, 90, 91), and the MICA-129 Met/Val dimorphism in α_2_ domain of the MICA protein (88, 92, 93).

## Conclusion

Despite some isolated conflicting results and sometimes scarce data, current research globally converges to the postulate that the NKG2D/NKG2DL axis is a new player in HCT. At the genetic level, most studies came to the conclusion that MICA mismatches between donor and recipients, either at amino acid position 129 or at the allelic level are deleterious and that whenever possible, an *MICA*-matched donor should be chosen for transplantation. Upregulation of both NKG2DLs and NKG2D during HCT have been demonstrated by various groups and associated with GVHD and GVL effects. Finally, recent data suggest a possible protecting role of anti-MICA antibodies *via* an antagonizing effect on sMICA that ultimately leads to promotion of GVL. Additional studies are, however, needed to definitely confirm these findings and to better understand the pathophysiological role of NKG2D and NKG2DLs in GVHD and GVL effect.

## Author Contributions

RC and SB wrote the manuscript and prepared the figures; WI and IA contributed to work reported in the manuscript.

## Conflict of Interest Statement

SB is the scientific founder and a (minority) shareholder in BIOMICA SAS.
